# Study on the Design Method of High-Resolution Volume-Phase Holographic Gratings

**DOI:** 10.3390/s24196493

**Published:** 2024-10-09

**Authors:** Shuo Wang, Lei Dai, Chao Lin, Long Wang, Zhenhua Ji, Yang Fu, Quyouyang Gao, Yuquan Zheng

**Affiliations:** 1 Changchun Institute of Optics, Fine Mechanics and Physics, Chinese Academy of Sciences, Changchun 130033, China; wangshuo206@mails.ucas.ac.cn (S.W.); dailei@ciomp.ac.cn (L.D.); linchao@ciomp.ac.cn (C.L.); wanglong_jixie@163.com (L.W.); jizh@ciomp.ac.cn (Z.J.); fuyang@ciomp.ac.cn (Y.F.); gaoquyouyang19@mails.ucas.ac.cn (Q.G.); 2 University of Chinese Academy of Sciences, Beijing 100049, China

**Keywords:** spectrometer, volume phase holographic grating, diffraction theory, grating design, diffraction performance, polarization

## Abstract

Volume-phase holographic gratings are suitable for use in greenhouse gas detection imaging spectrometers, enabling the detection instruments to achieve high spectral resolution, high signal-to-noise ratios, and high operational efficiency. However, when utilized in the infrared wavelength band with high dispersion requirements, gratings struggle to meet the demands for low polarization sensitivity due to changes in diffraction performance caused by phase delays in the incidence of light waves with distinct polarization states, and current methods for designing bulk-phase holographic gratings require a large number of calculations that complicate the balance of diffraction properties. To overcome this problem, a design method for transmissive bulk-phase holographic gratings is proposed in this study. The proposed method combines two diffraction theories (namely, Kogelnik coupled-wave theory and rigorous coupled-wave theory) and establishes a parameter optimization sequence based on the influence of design parameters on diffraction characteristics. Kogelnik coupled-wave theory is employed to establish the initial Bragg angle range, ensuring that the diffraction efficiency and phase delay of the grating thickness curve meet the requirements for incident light waves in various polarization states. Utilizing rigorous coupled-wave theory, we optimize grating settings based on criteria such as a center wavelength diffraction efficiency greater than 95%, polarization sensitivity less than 10%, maximum bandwidth, and spectral diffraction efficiency exceeding 80%. The ideal grating parameters are ultimately determined, and the manufacturing tolerances for various grating parameters are analyzed. The design results show that the grating stripe frequency is 1067 lines per millimeter, and the diffraction efficiencies of TE and TM waves are 96% and 99.89%, respectively. The diffraction efficiency of unpolarized light is more than 88% over the whole spectral range with an average efficiency of 94.49%, an effective bandwidth of 32 nm, and a polarization sensitivity of less than 7%. These characteristics meet the performance requirements for dispersive elements based on greenhouse gas detection, the spectral resolution of the detection instrument is up to 0.1 nm, and the signal-to-noise ratio and working efficiency are improved by increasing the transmittance of the instrument.

## 1. Introduction

The increase in greenhouse gas emissions contributes to global warming, seriously disrupts the ecological balance, and negatively affects human activities. Monitoring carbon dioxide (CO_2_), the most prevalent greenhouse gas, is particularly important [1]. Since the beginning of the twenty-first century, the United States, China, Europe, and other countries have conducted extensive research on greenhouse gas monitoring technology and launched a number of hyperspectral observation satellites. These include satellites capable of detecting greenhouse gases, such as Envisat, GF-5, and AIRS [2,3,4], as well as satellites specifically designed for CO_2_ detection, such as GOSAT, OCO-2, and TANSAT [5,6]. Grating-based imaging spectrometers are currently the main type of CO_2_ detection instrument [7,8,9], and grating is a key component that affects the performance of the spectrometer [10]. Traditional mechanical straight gratings are prone to ghost lines and low diffraction efficiency, which affects their ability to meet detection performance requirements. As a result, researchers have developed new types of gratings, such as immersion gratings, although these are complex to manufacture and expensive to produce [11,12,13]. In contrast, volume-phase holographic gratings (VPHGs) can improve the performance of spectrometers while significantly reducing the size and weight of the instrument, thereby reducing the cost of on-board satellites [14,15].

The concept of “holography” was first introduced by Gabor. Later, Denisyuk combined Lippmann’s “volume grating” and Gabor’s holography to invent “volume holography” [16]. VPHGs represent a fundamental form of volume holograms, offering advantages such as high diffraction efficiency, high signal-to-noise ratio, uniform dispersion, and low production costs [17]. VPHGs are widely used in fields such as optical communication [18,19], holographic displays [20,21,22,23], lasers [24], and imaging spectroscopy [25,26]. Diffraction theory plays a crucial role in the design of VPHGs. Currently, Kogelnik coupled-wave theory and rigorous coupled-wave analysis (RCWA) are widely used in the design of VPHGs [27,28]. There are two primary methods for designing VPHGs: 1. Using commercial software or custom programs based on RCWA theory. This approach involves extensive iterative computations, slow processing speed, and complex constraint settings, which hinder intuitive quantitative analysis [29]. For instance, VPHG achieved a maximum diffraction efficiency of about 97% in the HERMES high-resolution spectrograph project at the Australian Astronomical Observatory, but its bandwidth was approximately 14 nm [30]. 2. Using custom program design based on Kogelnik theory. Unfortunately, due to the approximations inherent in this theory, the design results can be inaccurate, leading to suboptimal grating performance. Precise quantitative analysis remains challenging [26,27]. For instance, high-line-density transmissive VPHG, designed by the Space Centre in Liège, Belgium, has a maximum diffraction efficiency of about 88% but exhibits poor polarization sensitivity [31]. VPHGs designed for small Bragg angles in the visible light range achieve high diffraction efficiency, low polarization sensitivity, and wide spectral range while maintaining a certain level of line dispersion [32]. In the infrared range, with a trade-off in the dispersion capability of the grating, high diffraction efficiency, low polarization sensitivity, and wide spectral range are attainable [33]. Alternatively, by considering only the TE or TM wave incidence, high diffraction efficiency and wide spectral range can be achieved. Analysis of design results for VPHGs reveals that current design methods are predominantly based on a single diffraction theory. Meeting the requirements for high spectral resolution within the CO_2_ detection wavelength range poses challenges in simultaneously optimizing diffraction efficiency, bandwidth, and polarization sensitivity.

To overcome the above limitations, this paper presents a design method for short-wave infrared transmission VPHGs for carbon dioxide detection applications. The method combines two diffraction theories (namely, Kogelnik coupled-wave theory and rigorous coupled-wave theory), analyzes the effects of different grating parameters on the diffraction performance, and develops an iterative optimization method. The initial parameter ranges are determined based on polarization sensitivity, diffraction efficiency. and Kogelnik theory. Subsequently, the grating parameters that meet the requirements are iteratively refined using the RCWA theory and constraints, and the optimal design of the grating is finally obtained. The designed grating was also analyzed for tolerance of manufacturing parameters. The final design results show that the center wavelength of the VPHG is 1.635 μm, and the diffraction efficiencies of the TE and TM waves are 96% and 99.89%, respectively. The effective bandwidths are 86 nm and 32 nm, respectively, and the diffraction efficiencies are more than 88% in the unpolarized light spectrum. The average diffraction efficiency is 94.49% and the polarization sensitivity is below 7%, which improves the detection accuracy of the instrument. The design method reduces the required iterative data, has high design accuracy and fast calculation speed, and effectively balances the various coupling properties of the grating. It also solves the problems related to polarization in infrared spectroscopy and produces gratings with high diffraction efficiency, wide bandwidth, and low polarization sensitivity, thus verifying the feasibility of CO_2_ detection using VPHG.

## 2. Fundamental Principles of VPHG

### 2.1. Records of VPHG

Holographic gratings are special primitive gratings in which the recorded light is all plane waves. They can be categorized as surface holographic gratings and body holographic gratings according to the thickness of the recording material, which is usually differentiated using the parameter *Q* as follows [34]:

(1) $$Q=\frac{2\pi \lambda d}{n_{2}\Lambda }$$

where *λ* is the wavelength of the recorded light, *d* is the thickness of the recording medium, *n*_2_ is the average refractive index of the material, and Λ indicates the grating period, with *Q* ≥ 10 as the criterion for VPHG classification.

**Figure 1:**
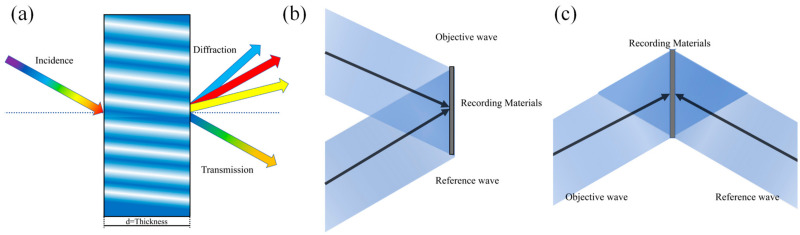
(**a**) Structure of the VPHG; (**b**) transmissive VPHG recording; (**c**) reflective VPHG recording.

VPHG is an element whose thickness is significantly greater than its grating period, and spectral separation is achieved by periodic spatial modulation of the refractive index. Figure 1a illustrates the structure of a VPHG formed by the interference of two recording beams incident on a photosensitive material [18]. Depending on the recording method, VPHG can be categorized as either transmissive or reflective. In transmissive-type VPHG, the recording beams are incident on the same side of the medium, as shown in Figure 1b, while in reflective-type VPHG, the recording beams are incident on the opposite side of the medium, as shown in Figure 1c.

### 2.2. Diffraction of VPHG

**Figure 2:**
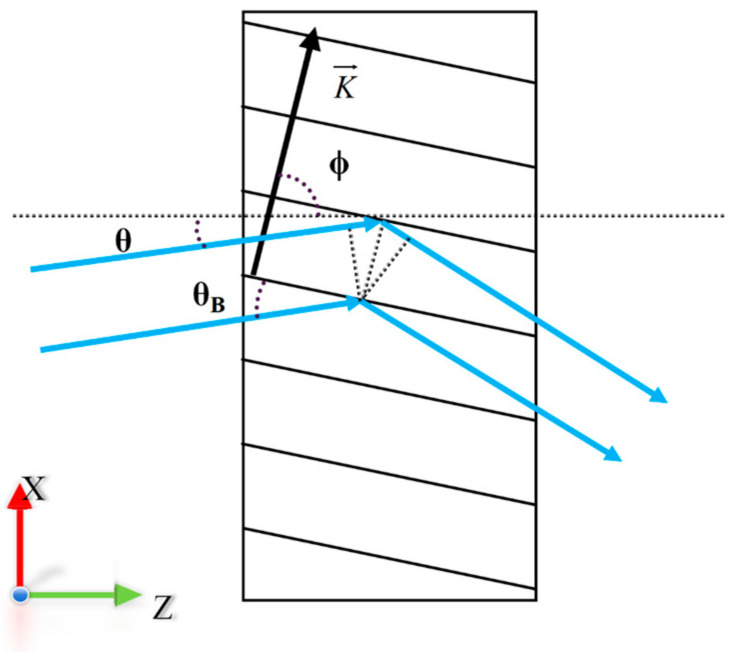
Bragg diffraction principle. (Blue arrows represent the incident and diffracted light, and black arrows represent the grating vector).

The diffraction of VPHG can be called “wavefront reproduction”, which is mainly based on the principle of Bragg diffraction, expressed as the phase length interference of scattered light equally spaced apart and with different striped surfaces in a particular direction. The principle of Bragg diffraction is shown in Figure 2, which can be expressed in terms of the following condition [27]:

(2) $$\cos\left(\phi -\theta \right)=\frac{K}{2\beta }$$

where *K* is the grating vector, *β* is the propagation constant, *ϕ* is the angle between the grating vector and the z-axis, and *θ* is the angle of incidence of the light within the grating medium. *K* and *β* are given by *K* = 2*π*/Λ and *β* = 2*πn*_2_/*λ_B_*, where Λ is the grating period of the VPHG, *n*_2_ is the average refractive index of the grating region, and *λ_B_* is the Bragg wavelength. The fabrication process is more complex when the grating fringes are tilted, leading to difficulties in controlling the grating constant and significant deviations between actual and theoretical performance. Therefore, this paper focuses on non-tilted fringe VPHGs, where *ϕ* = 90°, and the Bragg condition can be simplified to the following [34]:

(3) $$2n_{2}\Lambda \sin\theta_{B}=\lambda_{B}$$

where *θ_B_* is the Bragg angle, which represents the angle between the light within the grating and the grating planes.

**Figure 3:**
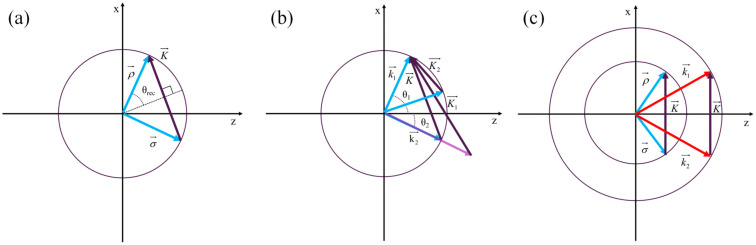
K-vector circle: (**a**) grating recording; (**b**) grating Bragg diffraction and diffraction deviating from Bragg conditions; (**c**) wavelength-shifted reconstruction principle.

The *K*-vector circle is a tool for qualitative analysis of VPHG. As shown in Figure 3a, the difference between the wave vectors of the two recorded light waves is equal to the grating vector. Figure 3b shows the Bragg diffraction of the grating as well as the diffraction with a deviation from the Bragg condition when the wave vector is larger than the recorded light wave vector or when the angle of incidence deviates from the recorded angle [19]. As can be seen, the Bragg condition is not fulfilled, and the diffracted intensity is reduced. This feature can be referred to as wavelength selectivity or angle selectivity of VPHG. The recording medium of VPHG is a photosensitive material that is generally more sensitive to visible light, but not to infrared light and cannot be recorded with the absorption spectrum of CO_2_. Figure 3c shows the variable wavelength reproduction principle of VPHG, that is, different wavelengths of diffracted light change the original Bragg angle, so that the vector difference between the incident light and the diffracted light is again equal to the recording grating vector and the diffracted light intensity is still a great value.

When a VPHG is used in CO_2_ detection imaging spectrometers, the grating parameters can be computed based on the application requirements. For example, in the case of a transmission VPHG with non-inclined grating fringes, the relevant equation can be derived from the grating equation, line dispersion formula, Bragg condition, the spectral resolution of the system, and the imaging lens parameters as follows:

(4) $$\tan\theta_{B}=\frac{cs\lambda_{B}}{2Af}$$

(5) $$\Lambda =\frac{\lambda_{B}}{2n_{2}\sin\theta_{B}}$$

where *cs* is the pixel size of the spectrometer detector, *A* is the spectral resolution, and *f* is the focal length of the imaging lens in the spectrometer. Given the parameters of the spectrometer, the grating period and Bragg angle can be calculated.

## 3. Comparison of Kogelnik Theory and RCWA Theory

### 3.1. Calculation of Two Diffraction Theories

**Figure 4:**
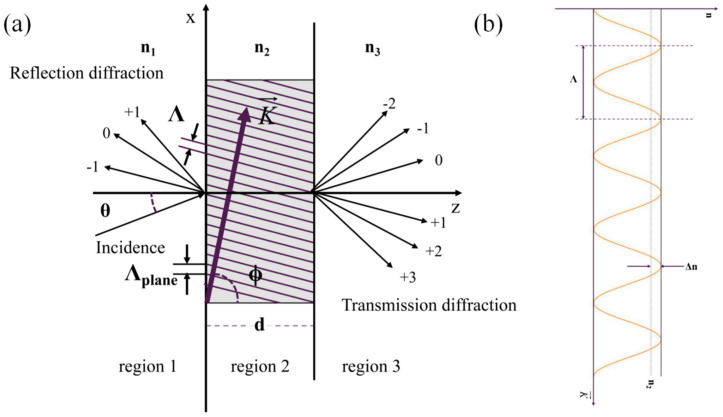
(**a**) Diagram of the principle of diffraction theory calculation; (**b**) types of refractive index modulation within the grating.

Diffraction theory is the main method for designing and analyzing the performance of gratings. Kogelnik coupled-wave theory and RCWA theory are commonly used to design and analyze VPHGs. The principle of diffraction theory calculation is given in Figure 4. The Kogelnik theory is a first-order two-wave theory that mainly calculates the diffraction efficiency by solving the differential equations of the incident and winding waves. In the calculation, the spatial modulation of the refractive index along the incident plane is expanded as follows [31]:

(6) $$n=n_{2}+\Delta n\cos\left(\vec{K}\cdot \vec{r}\right)$$

where Δ*n* is the modulation depth of the refractive index in the grating, and r is the radial vector. The cosine modulation of the refractive index couples the light waves in the grating region. This theory makes several assumptions in the calculation process. For instance, it assumes that there is only 0th- and 1st-order diffracted light in the grating region, the angle of incidence is assumed to be approximately equal to the Bragg angle, and the 2nd-order derivative of the electromagnetic field is neglected. Once these assumptions are met, the diffraction efficiency can be obtained by solving the coupled wave equations with boundary conditions and the generalized solution of Maxwell’s system of equations. For example, the diffraction efficiency equation for a non-tilted stripe transmitting VPHG when a TE wave is incident can be simplified to the following [35]:

(7) $$\eta =\frac{\sin^{2}{\left(\nu^{2}+\xi^{2}\right)}^{\frac{1}{2}}}{1+\frac{\xi^{2}}{\nu^{2}}}$$

(8) $$\nu_{TE}=\frac{\pi \Delta nd}{\lambda_{B}\cos\theta_{B}}$$

(9) $$\xi =\frac{\Delta \theta Kd}{2}-\frac{\Delta \lambda Kd}{8\pi n_{2}\cos\theta_{B}}$$

where *ν_TE_* is the coupling characteristics of TE waves within the grating, and *ξ* is the deviation from the Bragg condition.

The RCWA theory is a rigorous vector differential theory. For TE wave incidence, the grating is initially stratified along the thickness direction. The electromagnetic field of the incident light and the dielectric constant in the grating region are expanded into a Fourier series. Maxwell’s equations, together with the Rayleigh expansion, are then used to derive an expression for the electromagnetic field in each region. The coupled fluctuation equations are obtained by substituting the dielectric constant and the electromagnetic field in the fluctuation equations. A generalized solution of this system of equations is derived using the state variable method. Finally, the diffraction efficiency is calculated based on the boundary conditions of the electromagnetic field. The enhanced transmission matrix method is used in the calculation process to improve the calculation speed and convergence. The diffraction efficiency obtained is expressed as follows [28]:

(10) DE1i=Reξ1iξ10RiRi*

(11) DE3i=Reξ3iξ10TiTi*

where *DE*_1*i*_ and *DE*_3*i*_ are the diffraction efficiencies for the transmission and reflection regions, *i* is the diffraction order, *ξ_li_* is the propagation constant components along the grating thickness direction for different regions and diffraction orders, and *R_i_* and *T_i_* are the normalized amplitudes of the reflected and transmitted waves. When TM waves are incident, only the electromagnetic field expressions need to change, and other processes remain unchanged.

### 3.2. Theoretical Similarity Judgment

**Figure 5:**
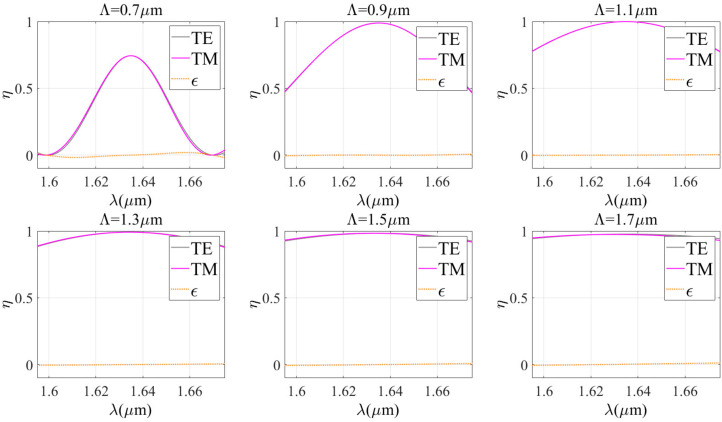
Comparison of the two theories for different grating periods.

**Figure 6:**
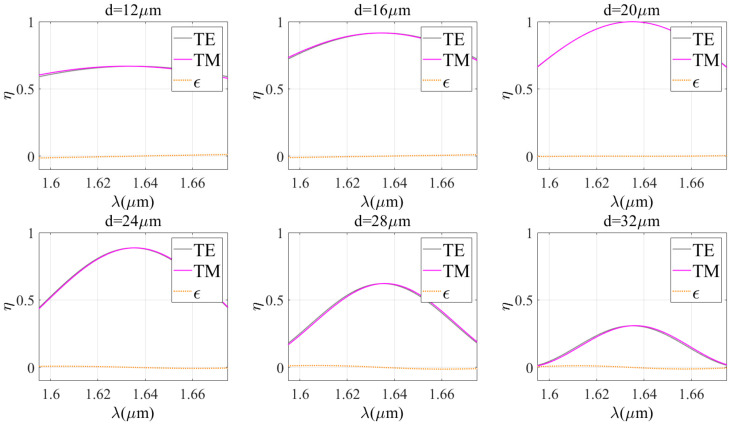
Comparison of the two theories for different grating thicknesses.

**Figure 7:**
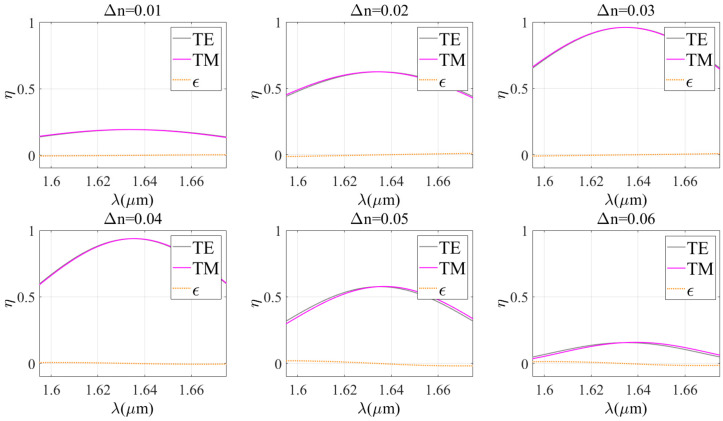
Comparison of the two theories for different refractive index modulations.

Kogelnik theory provides a simple calculation method and fast calculation speed, but the results may have limitations in accuracy. In contrast, the RCWA theory has high modeling accuracy and wide applicability, but its computational process is complex and time-consuming. The combination of these two theories enables us to combine the advantages of both theories and determine the optimal diffraction properties more effectively, faster, and more accurately. Therefore, first of all, it is very important to evaluate the applicability of each theory and verify the possibility of their combined use. Thus, the effect of wavelength on diffraction efficiency under TE wave incidence conditions is studied. The similarity between the theoretical curves is evaluated using the Spearman correlation coefficient and the overlap between the theoretical curves is evaluated using the Euclidean distance. Figure 5, Figure 6 and Figure 7 show a comparison between the two theories for different grating periods (Bragg angles), refractive index modulations, and grating thicknesses, while other parameters are kept constant.

The Spearman correlation coefficients and Euclidean distances of the two theories under different parameter conditions are given in Table 1. It is clear that the Euclidean distance between the two theories is always less than 0.5 under different conditions. When the grating period is less than 1.7 μm, the grating thickness is more than 12 μm, the refractive index modulation is less than or equal to 0.06, and the correlation coefficient is more than 0.9. This indicates that there is a very high correlation and approximation between the two theories. In these cases, the Kogelnik theory is applicable. Therefore, the results of Kogelnik theory are very close to the exact results when certain parameter conditions are met. It is suggested to combine the two diffraction theories in such a way that combining the advantages and disadvantages of the two theories leads to the optimal design of VPHG.

## 4. Analysis of VPHG Diffraction Performance

When a VPHG is used as a dispersive element, an important evaluation metric is the bandwidth, which is defined as the full width of the main lobe of the wavelength versus the diffraction efficiency curve. The expression for calculating bandwidth using Kogelnik Theory is

(12) $$\Delta \lambda =\frac{{\left(\pi^{2}-\nu^{2}\right)}^{\frac{1}{2}}\lambda_{B}^{2}}{\pi n_{2}d\tan\theta_{B}\sin\theta_{B}}$$

Using the above equation, the effect of grating parameter variation on its bandwidth can be analyzed. Another important evaluation index of the grating is the polarization sensitivity, which is mainly characterized by the difference in diffraction efficiency between TE and TM wave incidences. According to the Kogelnik theory, the diffraction efficiency of the TM wave is different from that of the TE wave, mainly due to the difference in the parameter *ν*, which is illustrated below:

(13) $$\nu_{TM}=\nu_{TE}\cos2\theta_{B}$$

where cos2*θ_B_* represents the difference in diffraction efficiency for different polarizations, also referred to as phase delay. When cos2*θ_B_* = 1, it indicates no polarization. However, since −90° < *θ_B_* < 90° and *θ_B_* = 0° do not meet the incidence conditions for non-tilted transmission VPHGs, the TE and TM wave diffraction cannot be identical. When analyzing the diffraction characteristics of VPHG using the Kogelnik theory, the results can be uncertain due to the approximation. Therefore, a rigorous simulation analysis based on RCWA theory is necessary to accurately assess the diffraction characteristics.

**Figure 8:**
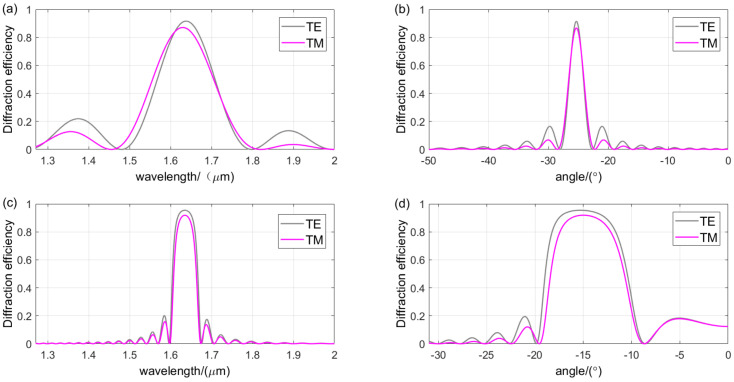
Comparison of the diffraction performance of the two types of gratings: (**a**) wavelength selectivity curves for different polarization states of the transmission-type VPHG; (**b**) angular selectivity curves for different polarization states of the transmission-type VPHG; (**c**) wavelength selectivity curves for different polarization states of the reflection-type VPHG; (**d**) angular selectivity curves for different polarization states of the reflection-type VPHG.

Figure 8 shows a performance comparison between transmission-type VPHG and reflection-type VPHG. Gelatin dichromate with a refractive index of 1.52 is usually chosen as the grating recording material [36]. The grating period of transmission VPHG is 1.2726 μm, the Bragg angle is 25°, the thickness is 22 μm, and the refractive index modulation is 0.04. The grating period of reflection VPHG is 2.078 μm, the Bragg angle is 15°, the thickness is 28 μm, and the refractive index modulation is 0.04. A comparison of bandwidths and angular bandwidths of the two grating types shows that transmission VPHG has a larger wave bandwidth, but smaller angular bandwidth. Although the reflective VPHG has better angular selectivity, its stronger wavelength selectivity limits its ability to cover the spectral range required for detection. In addition, the expansion and contraction of the gelatin layer during fabrication can complicate the control of the grating period and lead to significant performance deviations. Therefore, transmission VPHGs are more suitable as dispersive elements in spectrometers.

**Figure 9:**
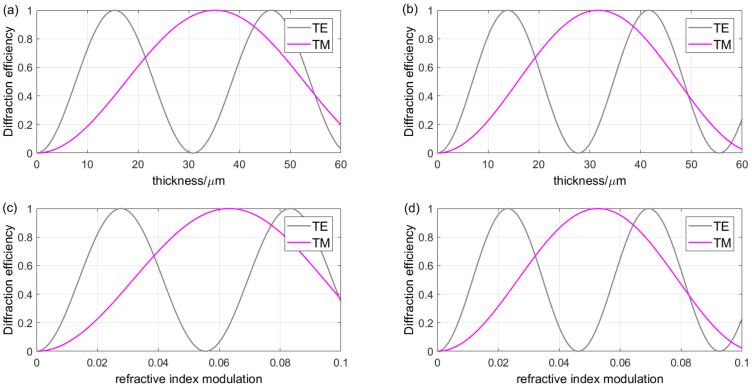
(**a**) The relationship between diffraction efficiency and grating thickness for different polarization states when Δ*n* = 0.045. (**b**) The relationship between diffraction efficiency and grating thickness for different polarization states when Δ*n* = 0.05. (**c**) The relationship between diffraction efficiency and refractive index modulation for different polarization states when *d* = 25 μm. (**d**) The relationship between diffraction efficiency and refractive index modulation for different polarization states when *d* = 30 μm.

VPHG is a thick-film optical element, typically with a thickness in the micrometer scale. The variation in diffraction efficiency with thickness when Λ = 1.0141 μm, *θ_B_* = 32.03°, and *λ_B_* = 1.635 μm while Δ*n* = 0.045 or 0.05 is shown in Figure 9a,b. As can be seen, the variations are sinusoidal, while the TM wave profile shows a phase delay compared to the TE wave profile. The refractive index modulation is a key factor in realizing VPHG diffraction. The variation of diffraction efficiency with refractive index modulation is shown in Figure 9c,d for different incident polarizations when Λ = 1.0141 μm, *θ_B_* = 32.03°, and *λ_B_* = 1.635 μm while *d* = 25 μm or 30 μm. Its effect on the diffraction characteristics is similar to that of the thickness. In addition, the variations of grating thickness and refractive index modulation do not affect the relative positions of the intersection points of the TE and TM wave diffraction efficiency curves.

**Figure 10:**
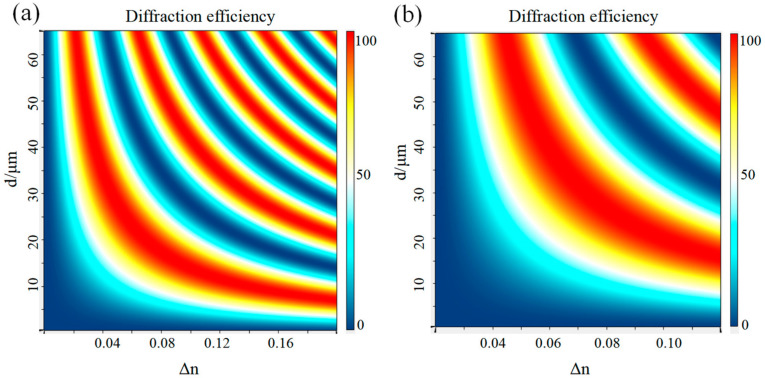
(**a**) The effect of d and Δ*n* on diffraction efficiency for TE wave incidence. (**b**) The effect of d and Δ*n* on diffraction efficiency for TM wave incidence.

**Figure 11:**
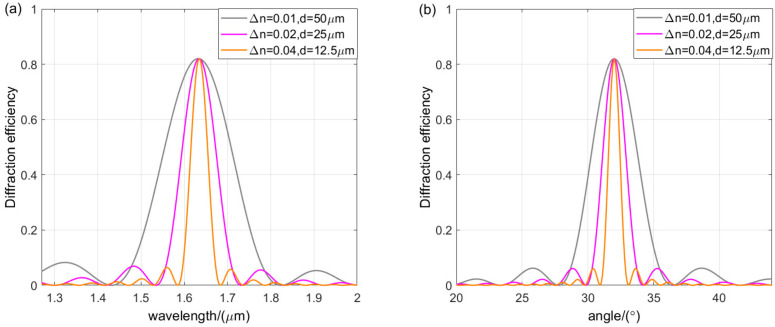
(**a**) Wavelength selectivity curves for different values of Δ*n* and *d* when Δ*n·d* is constant. (**b**) Angular selectivity curves for different values of Δ*n* and *d* when Δ*n·d* is constant.

Grating thickness and refractive index modulation have an important effect on the diffraction performance of gratings. Figure 10 shows a two-dimensional plot of Bragg wavelength diffraction efficiency with thickness and refractive index modulation while other parameters are kept constant. Peak overlaps can be observed at different polarization states, indicating a multiplicative factor relationship between the period and modulation curves of the diffraction efficiency with thickness for TE and TM waves. Figure 11 shows the wavelength and angular selectivity of the VPHG when the refractive index modulation and thickness are changed, but the product of the two remains constant. For a given product, increasing the refractive index modulation increases the bandwidth and angular bandwidth while the peak diffraction efficiency remains constant. On the contrary, increasing the grating thickness leads to a decrease in bandwidth and angular bandwidth, but does not change the peak diffraction efficiency.

**Figure 12:**
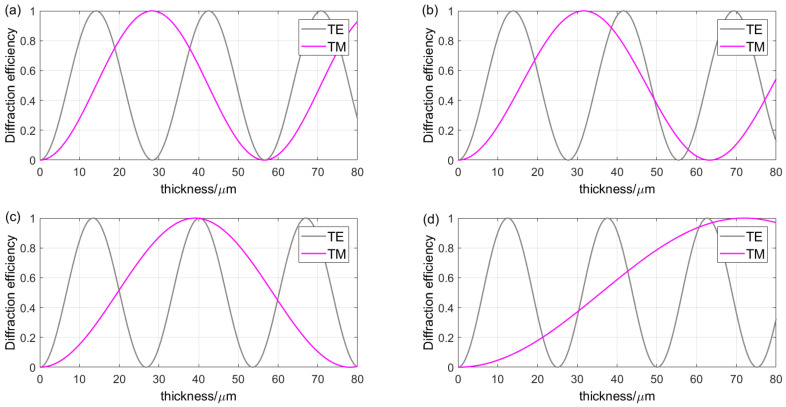
Thickness and diffraction efficiency curves for different periods and Bragg angles: (**a**) Λ = 1.0757 μm, *θ_B_* = 30°; (**b**) Λ = 1.0141 μm, *θ_B_* = 32.03°; (**c**) Λ = 0.9377 μm, *θ_B_* = 35°; (**d**) Λ = 0.8367 μm, *θ_B_* = 40°.

The grating period and Bragg angle of VPHG are key parameters in grating design, which have an important impact on the application of VPHG. Under different polarization incidences, *λ_B_* = 1.635 μm, Δ*n* = 0.05, and *d* range from 0 to 80 μm. Figure 12 shows the variation curves of the grating diffraction efficiency with grating thickness under different grating periods and Bragg angles. As the Bragg angle increases (the grating period decreases accordingly), the period of the TE wave profile becomes shorter while the period of the TM wave profile becomes longer. This then leads to an increase in the phase delay and a shift in the relative position of the intersection of the two. This indicates that the variation in the Bragg angle and grating period has a significant effect on the polarization sensitivity.

By using the formula of the Kogelnik theory and the simulation results of the RCWA theory, the effects of various parameters on VPHG diffraction performance can be analyzed in depth. The grating thickness is inversely proportional to the bandwidth. This is while the variation in thickness causes sinusoidal fluctuation in the Bragg wavelength diffraction’s efficiency but does not affect the relative position of the overlap point of the TE and TM wave diffraction efficiencies. This indicates that the thickness does not affect the optimization of polarization sensitivity. The refractive index modulation is proportional to the bandwidth, and its effect on the Bragg wavelength diffraction efficiency and polarization sensitivity is similar to that of the thickness. When the product of thickness and refractive index modulation is kept constant but the individual values are different, only the bandwidth is affected. Variations in grating period and Bragg angle can greatly affect the Bragg wavelength diffraction efficiency while having little effect on the bandwidth. But on the other hand, they can significantly change the relative position of the overlap points of the TE and TM wave diffraction efficiency. This makes grating period and Bragg angle valuable as constraints for optimizing polarization sensitivity.

## 5. VPHG Design Method and Result

### 5.1. Design Method

In spaceborne CO_2_ detection applications, the VPHG primarily receives non-polarized, shortwave infrared reflected light. Due to the low light intensity, the dispersive element should exhibit high diffraction efficiency and low polarization sensitivity, particularly in the peak channels of the CO_2_ absorption spectral features. Consequently, the design of VPHGs for CO_2_ detection should balance diffraction performance, ensuring that the diffraction efficiency, polarization sensitivity, and bandwidth achieve their theoretical design limits. Current design approaches typically rely on either Kogelnik theory or RCWA theory, each of which optimizes only one or two aspects of diffraction performance. Additionally, there is a lack of a clear design method to effectively address polarization sensitivity issues in VPHGs operating in the infrared spectrum. This paper introduces a hybrid design method for shortwave infrared transmissive VPHGs that integrates two diffraction theories to achieve balanced diffraction characteristics.

When incident light at the Bragg wavelength interacts with the VPHG and the bandwidth is disregarded, the key factors influencing the diffraction efficiency are grating thickness, refractive index modulation, period, and Bragg angle. But the polarization sensitivity is mainly affected by the grating period and Bragg angle. Therefore, by fixing the refractive index modulation and adjusting the period and Bragg angle, the required diffraction efficiency and polarization sensitivity can be achieved. It is necessary to calculate the Bragg angle range, which ensures that the diffraction efficiency is greater than 0.95 and the polarization sensitivity remains below 3% at the central wavelength. According to Kogelnik theory, the periods for diffraction efficiency and thickness curves for the TE and TM wave are π, and the peak diffraction efficiency positions are given by *mπ* + *π*/2, where *m* = 0, 1, 2…, etc. The size of a single period can be expressed as

(14) $$d_{TE}=\frac{\lambda_{B}\cos\theta_{B}}{\Delta n}$$

(15) $$d_{TM}=\frac{\lambda_{B}\cos\theta_{B}}{\Delta n\cos2\theta_{B}}$$

Thus, the periods of TE and TM waves can be related as multiples of each other, ensuring that the peak diffraction efficiency points of different periods are aligned. This alignment meets the requirements for both diffraction efficiency and polarization sensitivity under the given grating parameters. When the first period of the two curves approaches and the Bragg angle is less than 20°, the dispersion ability of the grating cannot meet the application requirements, as indicated by Equations (4) and (5). If the third period of the TE wave curve is close to the first period, or the second period is close to the third period, the relative grating thickness needed to achieve high diffraction efficiency and low polarization sensitivity becomes too large, hindering further bandwidth optimization. When the second period of the TE wave curve is aligned with the first period, the grating meets all performance requirements. An equation can then be found to achieve a diffraction efficiency of 0.95. That is,

(16) $$\sin^{2}\left(\nu_{TE}\right)=0.95$$

(17) $$\sin^{2}\left(\nu_{TM}\right)=0.95$$

**Figure 13:**
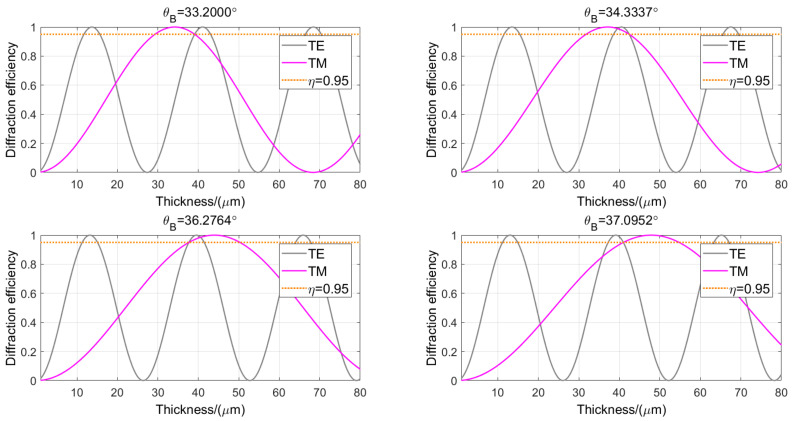
Four scenarios where the diffraction efficiency at the intersection points of the thickness and diffraction efficiency curves for different polarization states is equal to 0.95.

By simultaneously solving Equations (16) and (17), it is possible to determine the range of Bragg angles where the diffraction efficiency at the intersection of TE and TM wave curves is greater than or equal to 0.95. Figure 13 illustrates the relationship between the diffraction efficiency at these intersection points and the grating thickness, where the efficiency is 0.95. Within the Bragg angle range of 33.2° to 37.1°, suitable grating thickness and refractive index modulation can be identified to meet the requirements for both high diffraction efficiency and low polarization sensitivity.

This paper presents the design of a VPHG optimized for the CO_2_ absorption band that covers the spectral range from 1.620 μm to 1.650 μm with a central wavelength of 1.635 μm and requires a spectral resolution of 0.1 nm. For the Bragg angle range from 33.2° to 37.1°, the grating period, as determined by the Bragg condition, varies from 0.8916 μm to 0.9822 μm. The initial refractive index modulation is set at 0.04, with an initial grating thickness of 50 μm, which meets the design criteria for the combined diffraction theory. In the initial stage of design, the VPHG parameters are specified and the relationship between diffraction efficiency and thickness is determined for different polarization states. The thickness range should be more than twice the thickness period of the TE wave under initial conditions. Iterative adjustments in the Bragg angle range yield multiple sets of parameters (Λ, *θ_B_*, Δ*n*, *d*) that ensure the diffraction efficiency greater than 0.95 for both TE and TM waves. In the subsequent stage, these sets of parameters are refined with limitations on polarization sensitivity (less than 10%) and maximum bandwidth to determine the final parameter values (Λ, *θ_B_*, Δ*n*, and *d*). At this stage, the Bragg angle and grating period as well as the product of grating thickness and refractive index modulation are optimized. However, additional adjustments are made to extend the bandwidth. The final stage employs the maximum refractive index modulation value from the theoretical design constraints as a constraint to maximize bandwidth. The final VPHG parameters, as determined by the design program, are detailed in Table 2.

### 5.2. Verification of Grating Diffraction Performance

**Figure 14:**
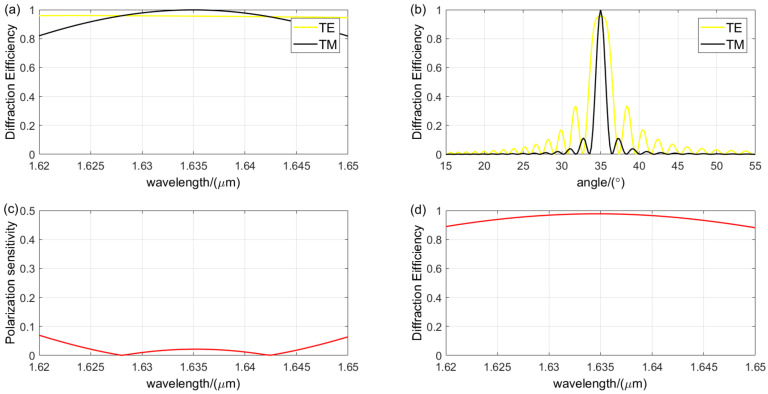
Analysis of the designed VPHG diffraction’s performance: (**a**) wavelength selectivity curves for different polarization states; (**b**) angular selectivity curves for different polarization states; (**c**) polarization sensitivity; (The red line indicates the value of polarization sensitivity.) (**d**) wavelength selectivity curve for non-polarized light. (The red line indicates the value of diffraction efficiency of unpolarized light).

The diffraction characteristics of the grating, as predicted by the RCWA theory for the designed parameters, are illustrated in Figure 14. The effective bandwidth is defined as the spectral range where the diffraction efficiency exceeds 80%. The designed VPHG features a central wavelength of 1.635 μm, with a maximum diffraction efficiency of 96% for TE waves and 99.89% for TM waves. In the spectral range, the diffraction efficiency for TE light is more than 94.5%, while for TM light it is more than 81.7%. The bandwidth and effective bandwidth for TE waves are 187 nm and 86 nm, respectively, while for TM waves, they are 116 nm and 32 nm. The diffraction efficiency remains above 90% for TE waves in the 1.6° bandwidth range and exceeds 80% for TM waves in the 0.8° bandwidth range. For non-polarized light, the diffraction efficiency exceeds 88%, with an average value of 94.49%. The polarization sensitivity is less than 7%. All diffraction characteristics of the grating meet the requirements for CO_2_ detection applications.

This study compares the diffraction performance of the designed VPHG with that of existing and applied gratings, such as the HERMES spectrograph at the Anglo-Australian Telescope (AAT), which utilizes VPHG across its four channels. For the 470.8 to 489.3 nm channel, the grating parameters are designed with an incidence angle of 67.2°, a line density of 2400 lp/mm, and a thickness of 4 μm. The diffraction efficiency for TE wave at the center wavelength is approximately 97%, while for TM wave it is around 54%. The TE wave bandwidth is limited to only 14 nm, leading to a high degree of polarization [30]. The infrared VPHG designed by Kaiser Optical for the MMIRS spectrograph in the MMT telescope has a line density of 290 lp/mm and an incidence angle of 13.8° [29]. Its wavelength range spans from 1500 to 1800 nm, achieving a center wavelength diffraction efficiency of 92% for non-polarized light, with edge spectral diffraction efficiencies of 76% and 91%. Although the diffraction efficiency and bandwidth are commendable, the grating period is excessively large, failing to meet the dispersion requirements of CO_2_ detection instruments. This comparison reveals that existing VPHG designs focus on polarization performance in specific aspects of the grating. In contrast, the design method proposed in this paper achieves a balance among diffraction efficiency, bandwidth, and polarization sensitivity. This method allows for targeted design based on application requirements, and the performance analysis of the design results further validates this advantage.

## 6. Comparison of Design Methods and Tolerance Analysis

### 6.1. Comparison of Other VPHG Design Methods

After completing the analysis of the diffraction characteristics of the designed VPHG, it is necessary to compare its diffraction performance with gratings designed solely using the Kogelnik theory and those designed using the RCWA theory to validate the advantages of the proposed design method.

**Figure 15:**
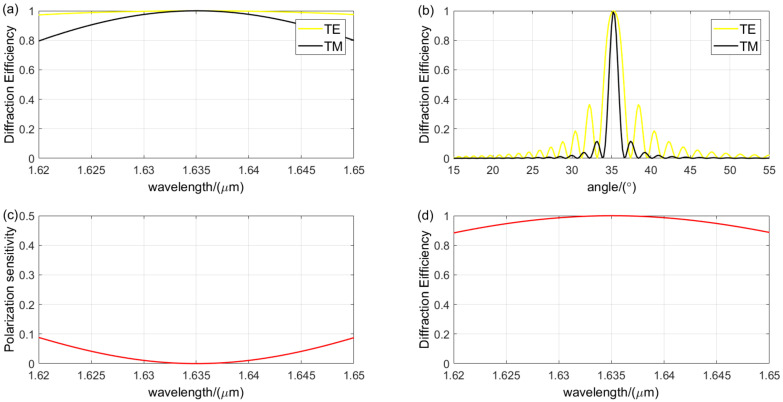
Diffraction performance analysis of VPHG designed using Kogelnik theory: (**a**) wavelength selectivity curves for different polarization states; (**b**) angular selectivity curves for different polarization states; (**c**) polarization sensitivity; (The red line indicates the value of polarization sensitivity). (**d**) wavelength selectivity curve for non-polarized light. (The red line indicates the value of diffraction efficiency of unpolarized light).

**Figure 16:**
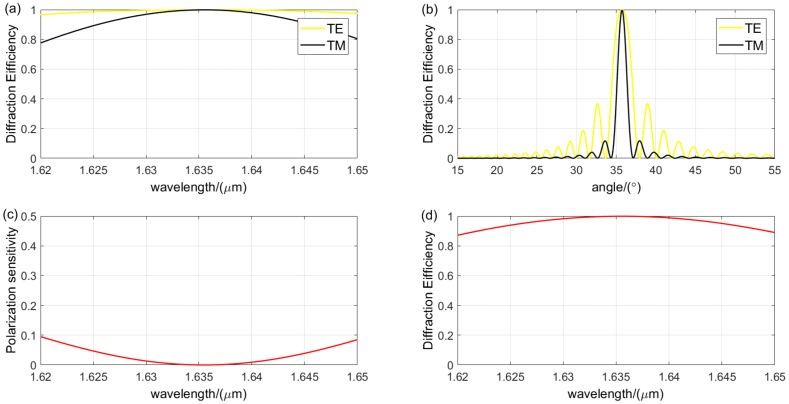
Diffraction performance analysis of VPHG designed using RCWA theory: (**a**) wavelength selectivity curves for different polarization states; (**b**) angular selectivity curves for different polarization states; (**c**) polarization sensitivity; (The red line indicates the value of polarization sensitivity). (**d**) wavelength selectivity curve for non-polarized light. (The red line indicates the value of diffraction efficiency of unpolarized light).

The design using either the Kogelnik theory or the RCWA theory primarily involves analyzing the diffraction characteristics of parameters to identify the grating period and Bragg angle where the second peak of TE wave coincides with the first peak of TM wave in the diffraction efficiency and grating thickness curves. Subsequently, the grating parameters are fine-tuned based on the results of the diffraction characteristics analysis to achieve the final design outcome. Figure 15 presents the analysis of the diffraction characteristics for the optimal grating parameters obtained using the Kogelnik theory, with a grating period of 0.9440 μm, a Bragg angle of 35.27°, and a grating thickness of 33.34 μm. The refractive index modulation is 0.06, achieving a diffraction efficiency of over 88.3% for non-polarized light across the spectral range, with an average diffraction efficiency of 95.92%. The TE wave exhibits a diffraction efficiency greater than 90% within a 1.4° angular bandwidth, while the TM wave exceeds 80% within a 0.4° angular bandwidth, and the polarization sensitivity is below 8.8%. Figure 16 illustrates the analysis of the diffraction characteristics for the optimal grating parameters obtained using the RCWA theory, with a grating period of 0.9330 μm, a Bragg angle of 35.20°, and a grating thickness of 33.5 μm. The refractive index modulation is also 0.06, resulting in a diffraction efficiency exceeding 87.1% for non-polarized light across the spectral range, with an average diffraction efficiency of 95.59%. The TE wave shows a diffraction efficiency greater than 90% within a 1.7° angular bandwidth, while the TM wave surpasses 80% within a 0.7° angular bandwidth, and the polarization sensitivity is below 9.5%.

Table 3 compares the diffraction performance of the VPHG obtained through three design methods. The data indicate that while VPHG designed solely using the Kogelnik or RCWA theory exhibits favorable diffraction performance under TE wave incidence, it fails to achieve a diffraction efficiency of over 80% for TM wave incidence within the spectral range of 1.620 to 1.650 μm, which is critical for greenhouse gas detection. Additionally, both the bandwidth and angular bandwidth do not reach their design limits, and the polarization sensitivity is higher than that of the results from the combined theoretical design. These two design methods primarily focus on the diffraction efficiency and polarization sensitivity at the center wavelength, neglecting a balanced optimization of the VPHG’s overall diffraction performance. This comparison suggests that a collaborative design approach using both theories is more suitable for high-resolution VPHG design in the field of CO_2_ detection.

### 6.2. Tolerance Analysis

**Figure 17:**
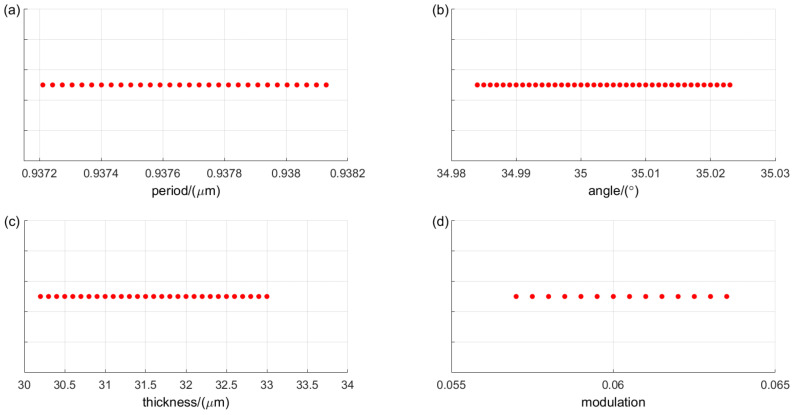
Results of tolerance analysis for different parameters of VPHG: (**a**) Analysis results of grating period accuracy; (**b**) Bragg Angle accuracy analysis results; (**c**) Accuracy analysis results of grating thickness; (**d**) Accuracy analysis results of the index modulation system.

In the actual manufacturing of volume phase holographic gratings, common fabrication errors include grating period errors, Bragg angle errors, grating thickness errors, and refractive index modulation errors. These errors can adversely affect grating performance, leading to decreased diffraction efficiency, reduced bandwidth, and poorer polarization sensitivity. Therefore, it is essential to analyze and establish reasonable tolerance values. A diffraction efficiency greater than 80% at the edge wavelengths within the spectral range is used as a selection criterion. Figure 17 illustrates the acceptable value ranges for each parameter of the VPHG, with each point representing a parameter value that meets the criteria.

Organizing the results of the tolerance analysis provides the tolerance ranges for grating in actual manufacturing, as shown in Table 4. The errors in grating thickness and refractive index modulation have relatively low sensitivity to diffraction performance, whereas errors in grating period and Bragg angle exhibit higher sensitivity.

## 7. Conclusions

In this paper, the design cycle and Bragg angle formulas for the application of VPHG in spectrometers are derived from the basic principles of VPHG. A diffraction efficiency calculation program is prepared based on two diffraction theories and the two theories are compared. By calculating the Spearman correlation coefficients and Euclidean distances for different grating parameters, the conditions for co-design using these theories are demonstrated. The effects of different grating parameters on the diffraction characteristics are analyzed using Kogelnik theory and diffraction property analysis. The constraints and iterative parameter adjustment methods are developed for the design of gratings. The design work is carried out according to the CO_2_ detection requirements. The initial Bragg angle and period range are calculated based on the Kogelnik theory. Constraints and iterative sequences are then defined based on the desired diffraction characteristics and parameter effects. The iterative optimization is then carried out using a rigorous coupled wave analysis theoretical procedure to design a transmission VPHG suitable for the CO_2_ detection band. Based on the requirements for grating diffraction efficiency, wave bandwidth, and polarization sensitivity, along with the design parameters for manufacturing tolerance analysis, the results confirm the feasibility of grating fabrication. The designed grating achieves diffraction efficiencies of 96% and 99.89% for the center wavelengths of TE and TM waves, respectively, and maintains diffraction efficiencies of 94.5% and 81.7% or greater across the entire spectral range. It also exhibits effective bandwidths of 86 nm and 32 nm, respectively, with polarization sensitivities below 7%. The diffraction efficiency of unpolarized light within the operational band exceeds 88.1%, with an average value of 94.49% and a center wavelength of 97.945 nm. The VPHG design method adopted in this paper requires a small amount of calculation and has high accuracy, which can meet the requirements of CO_2_ detection and provide balanced diffraction performance in the theoretical design. The proposed method can solve the problem of low polarization sensitivity and the requirement of high line density in the infrared band. With high diffraction efficiency, large effective bandwidth, and low polarization sensitivity, the gratings are expected to be applied in VPHG design in various fields. This work provides valuable theoretical guidance for the fabrication of future gratings.

## Figures and Tables

**Table 1:** Spearman correlation coefficients and Euclidean distances for different grating parameters.

Λ/μm	0.7	0.9	1.1	1.3	1.5	1.7
Spearman coefficient	0.9939	0.9999	0.9999	0.9996	0.9773	0.9019
Euclidean distance	0.3302	0.0673	0.0391	0.0847	0.1230	0.1795
d/μm	12	16	20	24	28	32
Spearman coefficient	0.9622	0.9957	1.0000	0.9990	0.9972	0.9954
Euclidean distance	0.2121	0.1754	0.0308	0.1747	0.2957	0.2545
Δn	0.01	0.02	0.03	0.04	0.05	0.06
Spearman coefficient	0.9887	0.9936	0.9988	0.9990	0.9863	0.9518
Euclidean distance	0.0804	0.2018	0.1454	0.1373	0.3976	0.3182

**Table 2:** VPHG design parameters.

Parameter	Value
Wavelength range	1.62 μm~1.65 μm
Bragg wavelength	1.635 μm
Groove density	1067 lp/mm
Bragg angle	35°
Refractive index modulation	0.06
Average refractive index	1.52
Grating thickness	32 μm
Diffraction order	−1

**Table 3:** Comparison of VPHG diffraction performance for different design methods.

Diffraction Performance	Collaborative Design	Kogelnik Theory	RCWA Theory
Diffraction efficiency at 1.620 μm for TE wave	95.90%	97.19%	96.63%
Diffraction efficiency at 1.650 μm for TE wave	94.54%	97.45%	97.42%
Diffraction efficiency at 1.620 μm for TM wave	81.96%	79.51%	77.62%
Diffraction efficiency at 1.650 μm for TM wave	81.70%	79.93%	80.46%
Average TE wave diffraction efficiency	95.49%	99.08%	98.93%
Average TM wave diffraction efficiency	93.48%	92.76%	92.25%
TE wave effective wave bandwidth	86 nm	70 nm	73 nm
TM wave effective wave bandwidth	32 nm	28 nm	29 nm
Maximum polarization sensitivity	0.0697	0.0884	0.0950

**Table 4:** VPHG tolerance range.

Parameter	Tolerance Range
Grating period	−0.42 nm~+0.5 nm
Bragg angle	−64″~+75″
Refractive index modulation	−0.003~+0.0035
Grating thickness	−1.8 μm~+1 μm

## Data Availability

The data that support the findings of this study are available from the corresponding author upon reasonable request.
